# LncRNA H19 inhibits ER stress induced apoptosis and improves diabetic cardiomyopathy by regulating PI3K/AKT/mTOR axis

**DOI:** 10.18632/aging.204256

**Published:** 2022-08-30

**Authors:** Sixuan Wang, Jun Duan, Jiangquan Liao, Yan Wang, Xiang Xiao, Lin Li, Yi Liu, Huan Gu, Peng Yang, Dongliang Fu, Jinhang Du, Xianlun Li, Mingjing Shao

**Affiliations:** 1 Department of Endocrinology, China-Japan Friendship Hospital, Beijing 100029, China; 2 National Integrated Traditional and Western Medicine Center for Cardiovascular Disease, China-Japan Friendship Hospital, Beijing 100029, China

**Keywords:** lncRNA H19, DCM, ERS, apoptosis, PI3K/AKT/mTOR, ROS, Nrf2

## Abstract

Objective: Extensive studies have shown that ERS may be implicated in the pathogenesis of DCM. We explored the therapeutic effects of lncRNAH19 on DCM and its effect on ERS-associated cardiomyocyte apoptosis.

Methods: C57/BL-6j mice were randomly divided into 3 groups: non-DM group (controls), DM group (DCM), and lncRNAH19 overexpression group (DCM+H19 group). The effect of H19 on cardiac function was detected. The effect of H19 on cardiomyocyte apoptosis and cardiac fibrosis in DM was examined. Differentially expressed genes (DEGs) and activated pathways were examined by bioinformatics analysis. STRING database was applied to construct a PPI network using Cytoscape software. The expression of p-PERK, p-IRE1, ATF6, CHOP, cleaved caspase-3, -9, -12 and BAX proteins in cardiac tissue was used to determine the ERS-associated apoptotic indicators. We established the HG-stimulated inflammatory cell model. The expression of p-PERK and CHOP in HL-1 cells following HG was determined by immunofluorescence labeling. The effects of H19 on ERS and PI3K/AKT/mTOR pathway were also detected.

Results: H19 improved left ventricular dysfunction in DM. H19 could reduce cardiomyocytes apoptosis and improve fibrosis *in vivo*. H19 could reduce the expression of p-PERK, p-IRE1α, ATF6, CHOP, cleaved caspase-3, cleaved caspase-9, cleaved caspase-12, and BAX proteins in cardiac tissues. Furthermore, H19 repressed oxidative stress, ERS and apoptosis *in vitro*. Moreover, the effect of H19 on ERS-associated apoptosis might be rescued by LY294002 (the specific inhibitor for PI3K and AKT).

Conclusion: H19 attenuates DCM in DM and ROS, ERS-induced cardiomyocyte apoptosis, which is associated with the activation of PI3K/AKT/mTOR signaling pathway.

## INTRODUCTION

Diabetic cardiomyopathy (DCM) is one of the most important and harmful complications in the diabetic population, which completely influences the quality of life of patients and mortality [1, 2]. Characterized by ventricular remodeling including ventricular hypertrophy (CH), DCM enforces an enormous affliction on patients and society [3, 4]. Myocardial fibrosis, endoplasmic reticulum stress (ERS) and myocardial cell death contribute to the progression of DCM [5–7].

The ER is a major cellular organelle that controls extensive cellular processes, and is highly susceptible to changes in homeostasis [8]. The homeostasis of the ER is a slightly modified event, in case freshly produced unfolded protein loads surpass the folding capability of the ER, the unfolded protein response (UPR) is triggered, resulting in ERS [9–11] ERS is a type of pressure reaction that induces cell death and impairs cellular homeostasis [12]. When ERS is persistent and powerful, cells activate a variety of adaptive processes in response to changes in protein folding, a phenomenon dubbed the unfolded protein response (UPR) [13, 14]. In the absence of pressure, the three branches of the UPR, protein kinase RNA similar to ER kinase (PERK), activating transcription factor-6 (ATF6), and inositol requirement protein 1 (IRE1), are held inactive by the ER membrane [12, 13]. Under ERS, the activation of PERK directly phosphorylates serine 51 of eukaryotic translation initiation factor 2α (eIF2α) to relieve the ERS, and activates transcription factor 4 (ATF4), which plays a critical role in accelerating cell apoptosis [14–16]. More importantly, ERS-stimulated PERK/ATF4 pathway activation mediates the pro-apoptotic transcription factor DNA-damage-inducible transcript 3 (CHOP), inducing cell death [16]. Extensive studies have shown CHOP activation plays a key role in cell apoptosis underlying ERS [17, 18].

Long non-coding RNAs (lncRNAs) are defined as RNA transcripts (>200 nucleotides in length) without protein-coding potential [19, 20]. They can influence gene expression by acting as transcriptional regulators in a manner unrelated to protein encoding [21–23]. It has been established that lncRNAs are involved in a variety of physiological processes that contribute to the pathophysiology of numerous cardiovascular disorders, including DCM [24, 25]. One crucial lncRNA in DCM is lncRNA H19 which produces a 2.3-kb non-coding mRNA and is conserved via matriarchal evolution [21, 22, 26]. It has been shown recently that H19 is closely associated with the regulation of cardiac hypertrophy, fibrosis and DCM [23].

The phosphatidylinositol 3-kinase (PI3K)/AKT/mammalian target of rapamycin (mTOR) pathway is a dominant cell signaling pathway implicated in many physiological conditions in mammals and participates in preventing cell death [27–29]. Several studies have shown that excessive activation of the PI3K/AKT/mTOR pathway may repress ERS and inhibit cell apoptosis [30], and the oxidative stress plays the intermediate link between PI3K signal and ERS, based on the mechanisms as oxidative stress and ROS, and furthermore, ROS inspire unfolded protein reaction, ERS, which are a vicious cycle, and PI3K-Nrf2 signals could indirectly inhibit ERS by suppressing ROS [31–34]. The intracellular signal transduction pathways play a critical role in DCM [35]. However, the molecular mechanism of the effect of H19 on the PI3K/AKT/mTOR pathway in DCM remains unknown and the relationship between H19 and ERS-induced cardiomyocyte apoptosis has not been investigated yet. In this paper, we investigated the effects of H19 on myocardial ERS and cardiomyocyte apoptosis *in vivo* and *in vitro* with DCM mice. Furthermore, the role of H19 in the PI3K/AKT/mTOR signaling pathway and ERS-induced cell apoptosis was examined.

## METHODS

### Bioinformatics analysis

GSE101585, GSE26887 and GSE124405 datasets were downloaded from the GEO database. The differential analysis between the normal group and DCM group was conducted with |logFC|>2 and *p* < 0.05 to obtain the differentially expressed lncRNAs and mRNAs. The R-based Bioconductor package data probe was used to explain the DEGs and enriched pathways in three datasets. The Bayesian method was used to screen differentially expressed ncRNAs and mRNAs. LncRRIsearch (http://rtools.Cbrc.jp/LncRRIsearch/) and RNARNA (http://rtools.cbrc.jp/cgi-bin/RNARNA/index.pl) were used to predict the interaction between lncRNAs and mRNAs. The public database miRWalk (http://mirwalk.umm.uni-heidelberg.de/) was used to predict and verify the target of mir-140-5p interaction, and map the binding site between mir-140-5p and PIK3CA.

### Mice

Male C57BL/6 mice weighing 20 ± 2 g were purchased from SKBEX Biotechnology Co., Ltd, and randomly into 3 groups: the control group (injected with PBS), Diabetic cardiomyopathy (DCM) group (injected with STZ and intramyocardial injection with 10^9^ lncRNA H19 empty lentivirus) and DCM+LncRNA H19 OE group (injected with STZ and intramyocardial injection with 10^9^ lncRNA H19 over-expression lentivirus), and the animal models of DCM were established based on fasting blood glucose from tail vein higher than 16.7 mmol/L tested by the contour glucose meter (Roche). Mice were maintained for 8 weeks. All animal operations comply with the regulations of the animal ethics committee of China-Japan Friendship Hospital (zryhyy 61-21-05-05).

### Mouse ultrasound

In this study, high-frequency ultrasound system VEVO 2100 and 30 MHz center frequency scanning head were used to detect the heart of mice. After anesthesia, the mice underwent two-dimensional echocardiography. The left ventricular end-systolic diameter (LVESd), left ventricular end-diastolic diameter (LVEDd), left ventricular ejection fraction (LVEF) and left ventricular shortening fraction (LVFS) were recorded. At the same time, each index was measured at least 3 cardiac cycles.

### Western blotting

Firstly, the total protein was extracted, and the protein concentration was detected by a protein concentration detection kit. The detected protein was added to the buffer solution and boiled for 10 min for denaturation. Then, sodium dodecyl sulphate polyacrylamide gel electrophoresis (SDS-PAGE) was carried out. After that, the protein sample was transferred to PVDF membrane at constant current of 300 mA, sealed with 0.5% skim milk for 2 h at room temperature, and incubated at 4°C with primary antibodies of p-PI3K (17366s, CST, 1:800), p-AKT (ab38449, Abcam, 1:1000), p-mTOR (ab109268, Abcam, 1:800), p-PERK (3179s, CST, 1:800), p-IRE1α (ab48187, Abcam, 1:800), ATF6 (24169-1-AP, Proteintech, 1:1000), CHOP (15204-1-AP, Proteintech, 1:800), cleaved-caspase-3 (ab214430, Abcam, 1:1000), cleaved-caspase-9 (ab2324, Abcam, 1:1000), cleaved-caspase-12 (ab8117, Abcam, 1:1000), Bax (ab182733, Abcam, 1:1200), Nrf2, NOX2, NOX4 (Abcam, 1:1000) and β-actin (66009-1-Ig, Proteintech, 1:5000). The PVDF membrane was removed from the antibody incubation box, immersed in TBST, and shaken with a shaker for 10 min, and these steps were repeated for 3 times. After incubation with secondary antibodies at room temperature, the density of protein bands was analyzed by software.

### Cultivation of mouse cardiomyocytes

HL-1 cells were evenly seeded in 6-well plates (1–2 × 10^5^ cells/mL), and aseptically cultured with low glucose or high glucose medium containing 10% fetal bovine serum in an incubator (5% CO_2_ v/v, saturated humidity, 37°C). The cultured cells were divided into 6 groups: low glucose group, low glucose + LncRNA H19 OE group, high glucose group, high glucose + LncRNA H19 OE group, high glucose + Thapsigargin group, high glucose + LncRNA H19 OE + Thapsigargin group as well as LY294002 treated groups (PI3K inhibitor, 10 μM) [36].

### Q-PCR

Trizol reagent (Invitrogen: 15596026) was used to extract the total RNA in myocardial tissue of each group, put 50 mg tissue into EP tube and grind it quickly, add 1ml Trizol reagent to lyse at room temperature for 5 min, after 12000 rpm high-speed centrifugation, collect the supernatant and add 200 μL mix chloroform evenly, stand at room temperature for 3 min, and centrifuge at 12000 rpm at 4°C for 10 min. The bottom sediment is the total RNA of the tissue. After vacuum drying, dissolve the RNA with DEPC water, measure the RNA concentration with a micro ultraviolet spectrophotometer (Thermo: a51119500c), and then reverse transcribe the RNA into cDNA with PrimeScript RT Master Mix (Takara: rr036a). The reaction condition is pre-denaturation at 98°C for 2 min; denaturation at 95°C for 10 s, annealing at 55°C for 30 s, extension at 72°C for 30 s and 40 cycles. Next, the expression levels of lncRNA H19 and mir-140-5p were detected by quantitative real-time polymerase chain reaction (RT qPCR) using TB green premix Ex Taq (Takara: rr420a). Internal parameters are unified with GAPDH, 2−ΔΔ. The relative expression of indexes was calculated by the CT method. LncRNA H19: forward primer 5′-CAACATCCGTAA-3′; Reverse primer 5′-CATCACCGGACCATGTCA-3′. miR-140-5p: forward primer 5′-CCCTATGTAGTTACGTCATGC-3′; Reverse primer 5′-TGTCCGGT-3′. GAPDH: forward primer 5′-GGGTCCCAGCTTAGGTTCAT-3′; Reverse primer 5′-CTCGTGGTTCACACCATCA-3′.

### Lentivirus infects cardiomyocytes

In the first 18–24 hours of lncRNA H19 NC and OE and KD lentivirus infection, the cells were placed in a 6-well plate (1 × 10^5^ cells/well) and cultured overnight. Upon reaching about 50% confluence, the cells were cultured with Polybrene 1 ml fresh medium instead of the original medium, added with an appropriate amount of virus suspension and incubated at 37°C. After 4 hours, 1 mL fresh medium was added to dilute Polybrene. The culture was continued for 24 hours, and the medium containing the virus was replaced with a fresh medium. After 48 hours of culture, the fluorescence expression was observed under an inverted fluorescence microscope.

### Immunofluorescence assay of cell climbing slice

The cells were seeded in a 24-well plate (5 × 10^4^ cells/well) with cell climbing slides overnight, and fixed in 4% paraformaldehyde. Immunofluorescence staining was carried on with Rabbit monoclonal anti-p-PERK (p-T982, ab192591, Abcam, 1:80) and mouse monoclonal anti-CHOP antibody (66741-1-Ig, Proteintech, 1:50) and corresponding second antibody with red/green fluorescence light were used, according to the previous method [37].

### Statistical analysis

Experiment data were represented as $\bar{\text{x}}\;\pm \;\text{s},$ and Graphpad Prism 6.0 software was used to perform *t*-test and one-way ANOVA.

## RESULTS

### H19 improved left ventricular dysfunction in DM

**Figure 1:**
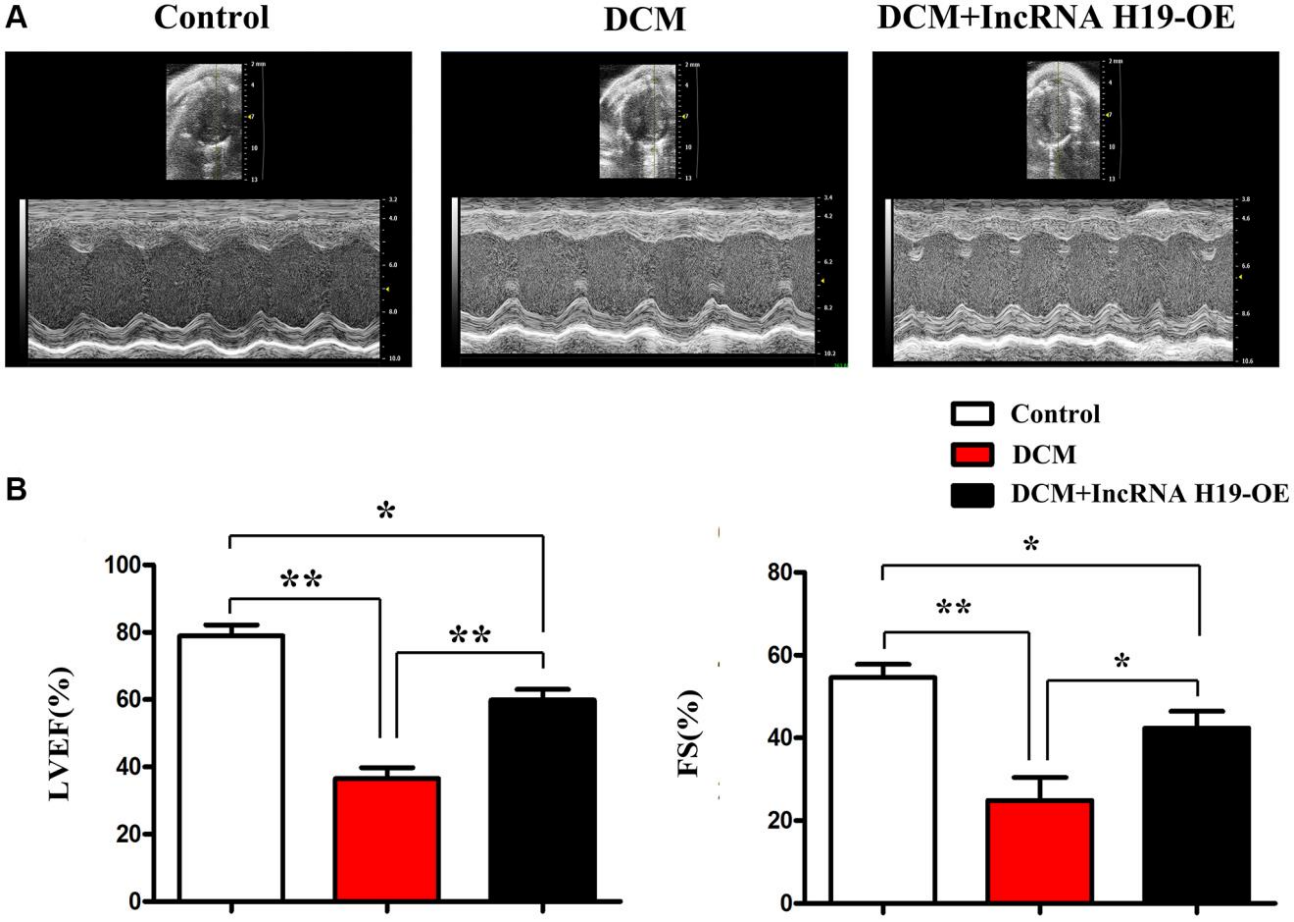
**H19 improved left ventricular dysfunction.** (**A**) Representative M-mode echocardiographic images. (**B**) LVEF and LVFS. Data are expressed as mean ± SEM. ^∗^*P* < 0.05. DCM vs. DCM+H19 group, *n* = 6/group.

DCM is characterized by cardiac dysfunction and damaged systolic function of the left ventricle [38, 39]. To examine the significance of H19 in heart function during DCM, we used lentivirus pcDNA-H19 to create H19 overexpression (OE) mice on a C57BL/6 background. In H19 OE and WT mice, streptozotocin (STZ) (50 mg/kg, ip) was injected continuously into the femoral vein for seven days to stimulate diabetes mellitus. The fasting blood glucose (FBG) above 350 mg/dL was considered DM. Ultrasonographic examination was conducted to examine the cardiac systolic function, in cardiac tissue after DM generation. Compared to the control mice, DCM mice exhibited significant cardiac dysfunction, as suggested by the decrease in LVEF and FS, while H19 effectively improved cardiac systolic function (Figure 1A). Furthermore, we found that the H19 administration efficiently improved the cardiac systolic function as implied by the enhanced LVEF and LVFS compared with the DCM group (*P* < 0.05, Figure 1B). And moreover, we investigated the empty lentivirus/NC had no significant influences on the progression of DCM vs. single STZ-induced DCM, data were showed on Supplementary Figure 1, these experiments were designed to avoid or determine the empty lentivirus had no significant effects on the DCM.

### H19 reduced cardiomyocyte apoptosis and cardiac fibrosis in DM

**Figure 2:**
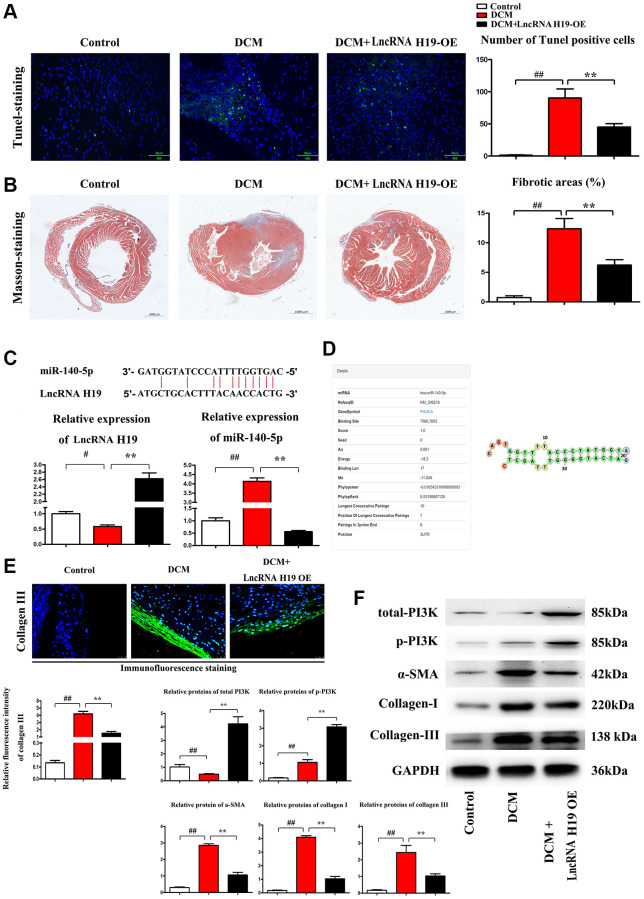
**H19 reduced cardiomyocyte apoptosis and cardiac fibrosis in DM.** (**A**) TUNEL staining was used to examine the apoptosis of cardiomyocytes. (**B**) Masson's trichrome staining was used to investigate the involvement of H19 in cardiac fibrosis in myocardial tissue. (**C**) The binding site of lncRNA H19 and miR-140-5p and relative expression of lncRNA H19 and miR-140-5p levels were determined by Q-PCR in each group. (**D**) Binding site of mir-140-5p to PIK3CA. (**E**) Immunofluorescence intensity of collagen III in each group. (**F**) The protein levels of p-PI3K, t-PI3K, a-SMA, and collagen-I/III in each group. ^#^*P* < 0.05; ^##^*P* < 0.01 vs. Sham group; ^*^P < 0.05; ^**^P < 0.01 vs. DCM group. between groups.

Pathological changes in DCM include cardiomyocyte apoptosis, ERS, myocardial fibrosis and inflammation. [40] TUNEL labeling was used to determine the apoptosis of cardiomyocytes in three groups of mice. We discovered that H19 therapy significantly lowered the number of TUNEL-positive (green) cells as compared to the DCM group (Figure 2A). The percentage of apoptotic cells grew dramatically in both DCM and DCM+H19 mice, and the percentage of apoptotic cells in the DCM+H19 mice was lower than that in the DCM group (Figure 2A, *P* < 0.05). There is evidence that myocardial fibrosis is proposed as a prospective contributor in the pathogenesis of DCM [41]. Following that, we used Masson’s trichrome staining to determine whether H19 is involved in cardiac fibrosis in myocardial tissue. The findings indicated that abundant collagen deposition and chamber dilation occurred in the myocardium of DCM mice. According to this comparison, DCM+H19 mice exhibited significantly reduced chamber dilation, interstitial fibrosis, and collagen deposition compared with DCM group. Moreover, the measurement of myocardial fibrosis areas exposed that myocardial fibrosis was also decreased in DCM+H19 group compared with that in DCM group (Figure 2B, *P* < 0.05). These findings suggested that H19 may repress apoptosis of cardiomyocytes and reduce cardiac fibrosis in DM. The relative expression of lncRNA H19 and miR-140-5p are shown in Figure 2C. H19 negatively modulated the expression of miR-140-5p determined by Q-PCR. Moreover, miR-140-5p could bind to PI3K (Figure 2D) thus the total PI3K and p-PI3K were tested by Western blotting. The results showed that the H19 overexpression resulted in the decreased expression of miR-140-5p (tested by Q-PCR) and increased the total PI3K and p-PI3K levels (tested by Western blotting) and the down-stream proteins, α-SMA, collagen-I/III were correspondingly decreased compared with sham and model groups (Figure 2F). Immunofluorescence staining was conducted on myocardial fibrosis-associated marker collagen III and H19 significantly suppressed the expression of collagen III (Figure 2E). And moreover, we further investigated the relationships between lncRNA H19, miR-140-5p when infected with lncRNA H19 NC, OE and KD group and we exhibited the relative expression levels of H19 and miR-140-5p showed opposite trend, and H19 over-expression showed significant increased activation or phosphorylated PI3K as well as raised expression of total-PI3K protein levels, and H19 KD showed the contrary results vs. H19 OE group, and data were exhibited in Supplementary Figure 2, these results indicated the H19 could Spongy with miR-140-5p, resulting into the no function of miR-140-5p, and considering the miR-14-5p degrade the PI3K mRNAs, therefore, we exhibited the H19-miR-140-5p axis could regulate PI3K protein expression.

### Overview of lncRNAH19 and PI3K expression in different DCM datasets

**Figure 3:**
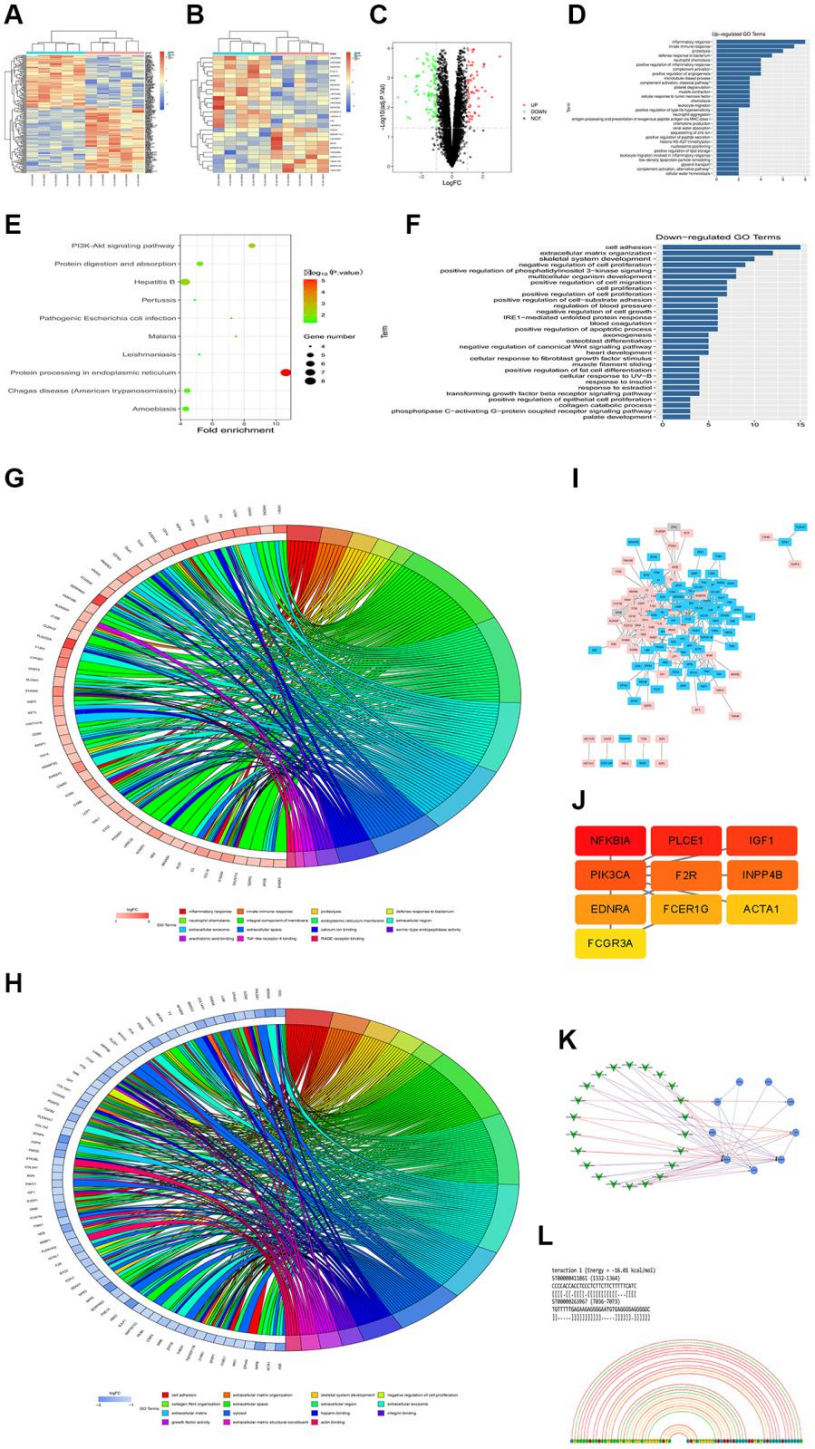
**Overview of lncRNAH19 and PI3KCA expression in different DCM datasets.** GSE101585, GSE26887 and GSE124405 were selected and downloaded from GEO database to identify the DEGs between DCM tissues and corresponding controls. (**A** and **B**) The heatmaps were screened out to illustrate the DEGs in DCM and corresponding controls using 124405 and GSE26887. (**C**) A volcano map was plotted to illustrate the DEGs using GSE101585. (**D**) GO enrichment analysis of abnormal regulation DEGs using GSE101585. (**E**) KEGG enrichment analysis of abnormal regulation DEGs using GSE101585. (**F**) GO biological processes analysis for the DEGs from GSE124401. (**G** and **H**) GO and KEGG enrichment analysis of abnormal regulation DEGs using GSE124405. (**I**) The PPI network of DEGs using GSE26887. (**J**) The top 10 hub genes in the PPI network were screened by Cytoscape plugin CytoHubba. (**K**) The Cytoscape co-expression analysis between differently expressed mRNAs and lncRNAs. (**L**) lncRNA-RNA interaction prediction.

Two gene expression profiles (GSE101585, GSE26887 and GSE124405) were selected and downloaded from GEO database to identify the differentially expressed genes (DEGs) between DCM tissues and corresponding controls. The differently expressed lncRNAs and mRNAs with *P* < 0.05 and |Log (FC)|>2 were identified in the three datasets. A heatmap was depicted to show the distribution of differently expressed lncRNAs (up- and down-regulated) between DCM and controls according to the data from GSE124405, including lncRNA H19 (Figure 3A). Moreover, another heatmap based on GSE26887 was plotted (Figure 3B), with 237 up-regulated and 249 down-regulated differently expressed mRNAs. Most importantly, a volcano plot was depicted to visualize the differently expressed lncRNAs between DCM and controls from the data in GSE101585 (Figure 3C). Additionally, lncRNA H19 was among the up-regulated DEGs.

To further understand the biological functions of all the DEGs, GO and KEGG pathway enrichment analyses were carried out. According to the DEGs from GSE101585, it was found that the DEGs were enriched in the biological processes, including positive regulation of the apoptotic process, regulation of phosphatidylinositol 3-kinase signaling, IRE1-mediated unfolded protein response, phospholipase c-activating G-protein coupled receptor signaling pathway, and cell proliferation (Figure 3D). The enrichment analysis of KEGG pathway showed that DEGs were noticeably enriched in the PI3K-Akt signaling pathway, Protein processing in the endoplasmic reticulum, and protein digestion and absorption (Figure 3E). Similarly, GO biological processes for the DEGs from GSE124401 are shown in Figure 3F. Furthermore, according to the data from GSE124405, GO and KEGG pathway enrichment analysis exposed that most DGEs were associated with inflammatory response, innate immune response, cell adhesion, collagen fibril organization, extracellular matrix, growth factor activity, and integral component of membrane signaling pathways (Figure 3G and 3H).

To assess the interaction of these DEGs in DCM, a PPI network was constructed using Cytoscape software utilizing data from GSE26887 using the STRING database (Figure 3I). Additionally, we use Cytoscape’s CytoHubba to obtain the hub genes, the 10 genes with the highest scores, including PIK3CA, are regarded as hub genes (Figure 3J). According to the Cytoscape co-expression analysis of differentially expressed mRNAs and lncRNAs shown in Figure 3K, the PI3KCA gene plays a critical role in regulating lncRNAs. In addition, the online database LncRRIsearch (http://rtools.cbrc.jp/LncRRIsearch/) and RNARNA (http://rtools.cbrc.jp/cgi-bin/index.cgi) were used to predict the interaction between lncRNAs and mRNAs. As shown in Figure 3L, among the identified target mRNAs, PI3KCA(PI3K) was chosen as a potential target of lncRNA H19. From our point of view, H19 might be associated with PI3K in DCM development and there is a need for further investigation.

### Effect of H19 on ERS-associated apoptosis markers and PI3K/AKT/mTOR -Nrf2 pathway in DM

**Figure 4:**
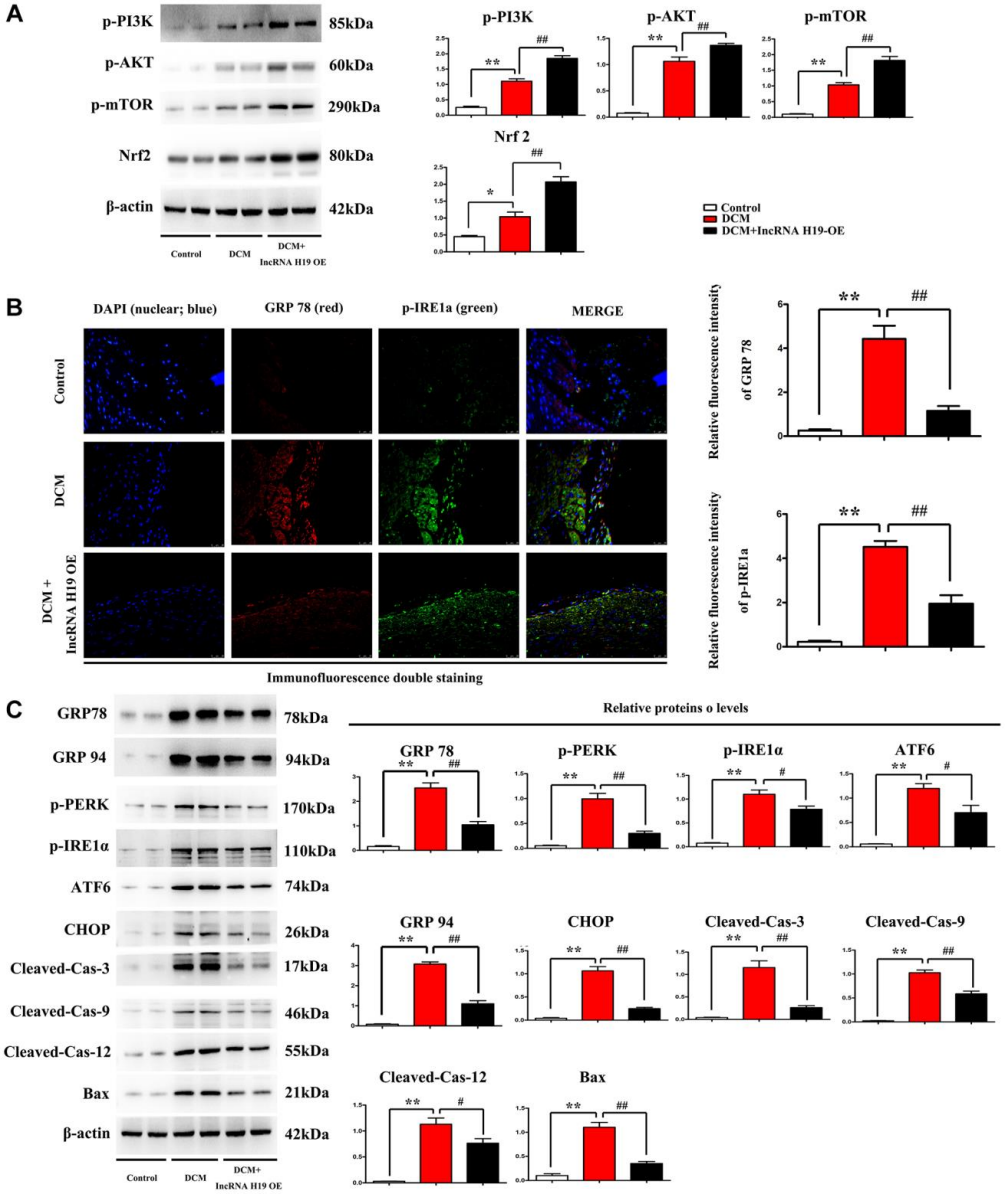
**Effect of H19 on ERS-associated apoptosis markers and PI3K/AKT/mTOR pathway in DM.** (**A**) Protein bands exposed the expression of p- PI3K, AKT and mTOR, Nrf2 in each group. (**B**) Immunofluorescence intensity of GRP78 and p-IRE1a in each group. (**C**) protein levels of p-PERK, p-IRE1α, ATF6, CHOP, cleaved caspase-3, cleaved caspase-9, cleaved caspase-12, and BAX proteins in all groups. ^#^*P* < 0.05; ^##^*P* < 0.01 vs. DCM group; ^*^P < 0.05; ^**^P < 0.01 vs. sham group, *n* = 6/group.

Western blotting was performed to evaluate the protein expression of the markers of ERS-associated apoptosis in cardiac tissues from the three groups. Compared to the control group, DCM group exhibited significantly enhanced protein expression levels of p-PERK, Nrf2, p-IRE1α, ATF6, CHOP, cleaved caspase-3, cleaved caspase-9, cleaved caspase-12, and BAX, while H19 reversed the up-regulation of these protein levels in cardiac tissues (Figure 4A). The results implied that the high glucose increased the ERS-induced cardiomyocyte apoptosis, which was noticeably reversed by H19. Furthermore, we assessed the phosphorylation of PI3K, AKT and mTOR as well as Nrf2. Similarly, the phosphorylation levels of PI3K, AKT and mTOR were slightly increased in DCM mice compared to control group and significantly enhanced by H19 (Figure 4A). These findings were confirmed by Western blotting, which revealed a significant increase of proteins expression in p-PI3K, p-AKT and p-mTOR in DCM+H19 group compared with those in the control group or DCM group (*P* < 0.05), consistent with decreased unfolded protein reaction (GRP78 and 94) as well as ERS (p-PERK, p-IRE1a, ATF6, CHOP) and apoptotic proteins (cleaved-caspase-3-9/-12 and Bax) tested by Western blotting (Figure 4C) or immuno- fluorescence staining (Figure 4B). To sum up, these findings suggested that H19 suppressed ERS-induced cardiomyocyte apoptosis by elevating the activation of the PI3K/AKT/mTOR pathway in DM.

### H19 represses ERS *in vitro*

**Figure 5:**
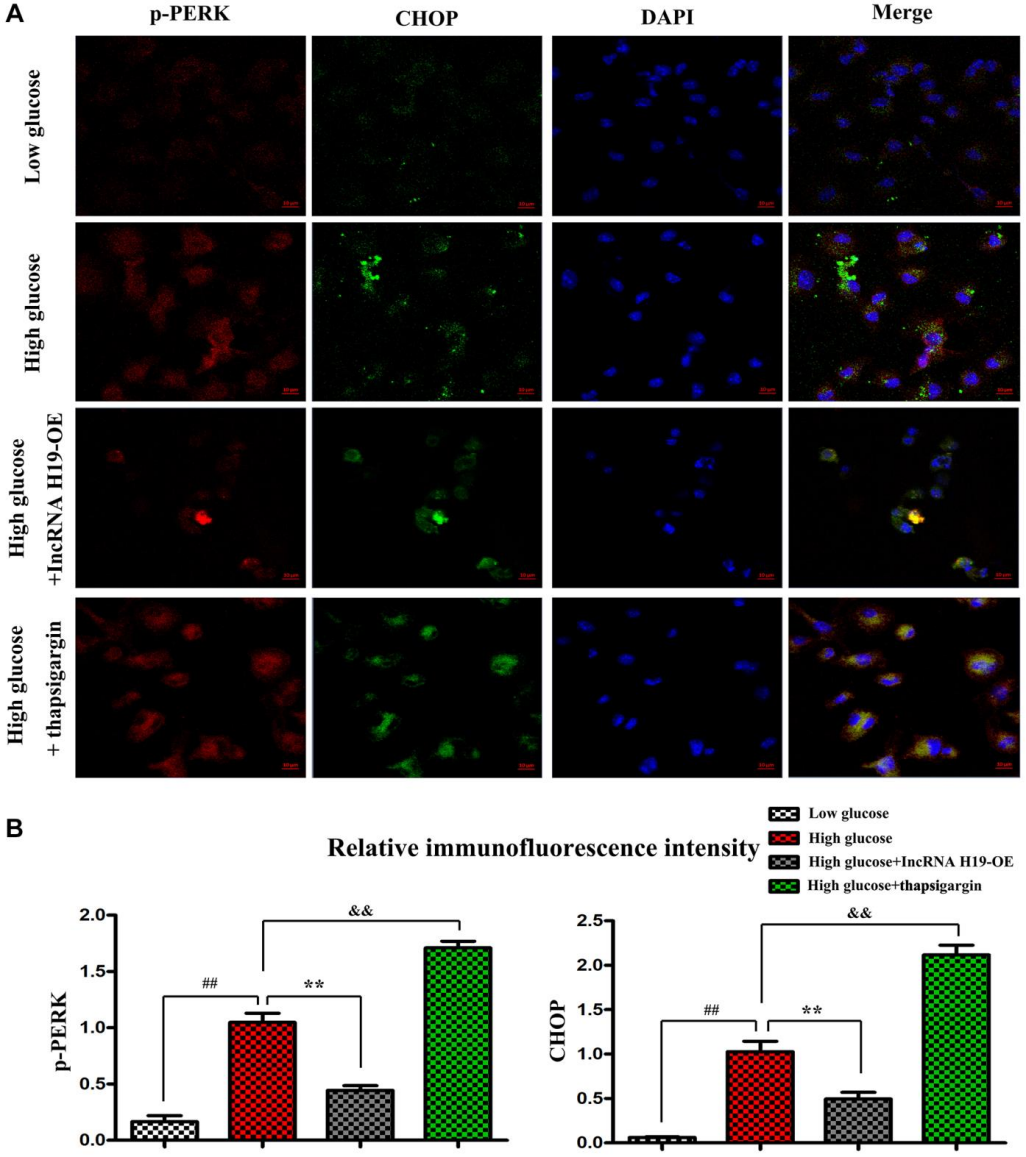
**H19 repressed ERS *in vitro*.** (**A**) Immunofluorescence staining exposed the expression of p-PERK and CHOP in four cell groups. (**B**) The statistical results of relative fluorescence intensity demonstrated the protein expression of p-PERK and CHOP in four cell groups. ^##^*P* < 0.05 Low glucose vs. High glucose. ^**^P < 0.05 High glucose+H19 OE vs. High glucose. ^&&^*P* < 0.05 High glucose vs. High glucose+ Thapsigargin, *n* = 3/group.

Research has shown that an ERS-induced increase of CHOP may trigger cell apoptosis and plays a critical role in regulating the fate of cells under ERS [42]. In the next tests, we used immunofluorescence staining to determine the effect of H19 on ERS in cardiomyocytes. Thapsigargin is a well-known ERS inducer that has been successfully used in a variety of cell types [43]. After pretreatment with lentivirus pcDNA-H19, HL-1 cells were induced with HG for another 24 hours and p-PERK and CHOP were identified with immunofluorescence staining. As shown in Figure 5A, the tendency was similar to that in Western blotting. HG stimulation significantly upregulated the expression of p-PERK and CHOP, whereas H19 markedly reversed the HG-induced downregulation of p-PERK and CHOP. In addition, p-PERK and CHOP were vastly enhanced in Thapsigargin-treated HL-1 cells. Compared to the cells in control group, the p-PERK and CHOP positive areas were significantly increased after HG treatment, while treatment with H19+HG could increase the areas in cardiomyocytes (Figure 5B, *P* < 0.05). Moreover, the cells in HG+ Thapsigargin group showed markedly increased p-PERK and CHOP expression compared to the HG group (*P* < 0.05). These findings suggested the upregulation of CHOP and p-PERK in HL-1 cells under HG stimulation, and the effect was decreased when cells were pretreated with H19.

### H19 attenuates ERS-induced apoptosis and activates the PI3K in HL-1 cells after high glucose stimulation

**Figure 6:**
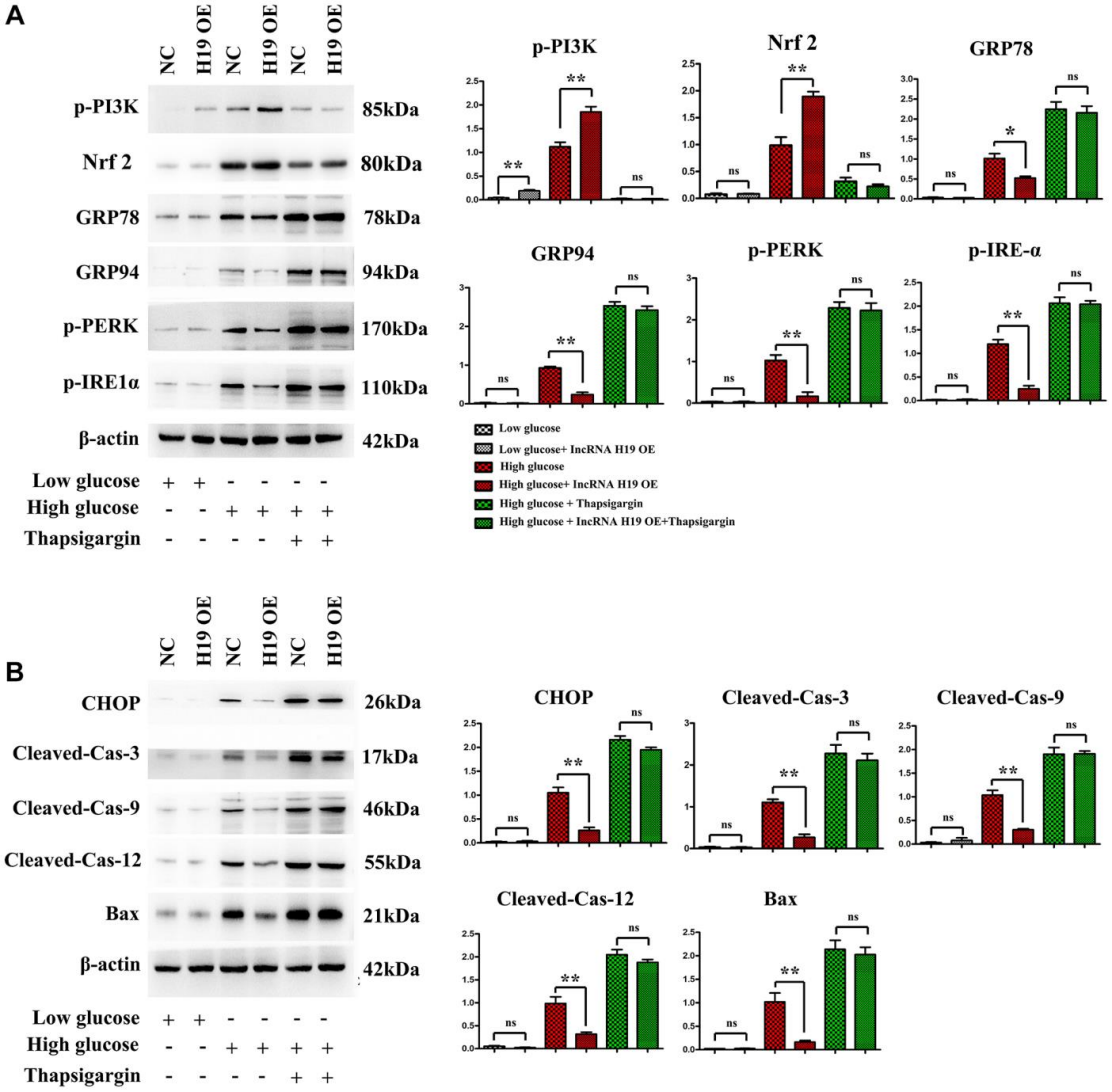
**H19 attenuated ERS-induced apoptosis and activated PI3K in HL-1 cells after HG.** (**A**) Protein bands exposed the expression of p-PI3K, Nrf2, p-PERK, p-IRE1α, GRP 78 and GRP94 proteins in four cell groups. (**B**) Protein bands exposed the expression of CHOP, cleaved caspase-3, cleaved caspase-9, cleaved caspase-12, and BAX proteins in four cell groups, *n* = 3/group.

The PI3K/AKT/mTOR pathway is a critical cell signaling pathway in regulating ERS-induced cell death [44], consequently, it is hypothesized that H19 can protect cardiomyocytes against ERS-induced cell apoptosis. Western blotting was used to evaluate the protein expression of p-PI3K, p-PERK, p-IRE1α, GRP 78 and GRP94. Compared with the control cell group, the p-PERK, p-IRE1α, GRP 78 and GRP94 levels were increased in the HG group and decreased in the HG+H9 pre-treatment group (Figure 6A, *P* < 0.05). Furthermore, Western blotting demonstrated that p-PI3K, p-PERK, Nrf2, p-IRE1α, GRP 78 and GRP94 protein levels were also markedly increased in HG+ Thapsigargin-treated cells compared with HG cell group (Figure 6A, *P* < 0.05). In addition, compared with the control or HG group, the phosphorylated p-PI3K was increased in the HG+H19-treated cell group (Figure 5B). As shown in Figure 6B, the protein expression levels of CHOP, cleaved caspase-3, cleaved caspase-9, cleaved caspase-12, and BAX were increased in the HG group and decreased in the HG+H9 pre-treatment group (Figure 6B). They were also marked increases in HG+Thapsigargin group compared with HG cell group (Figure 6B, *P* < 0.05). These findings suggest that H19 attenuates ERS-induced apoptosis and activates PI3K in HL-1 cells after HG.

### H19 suppressed ROS, ERS by activating PI3K-Nrf signals and these roles were corrected by PI3K specific inhibitor LY294002

**Figure 7:**
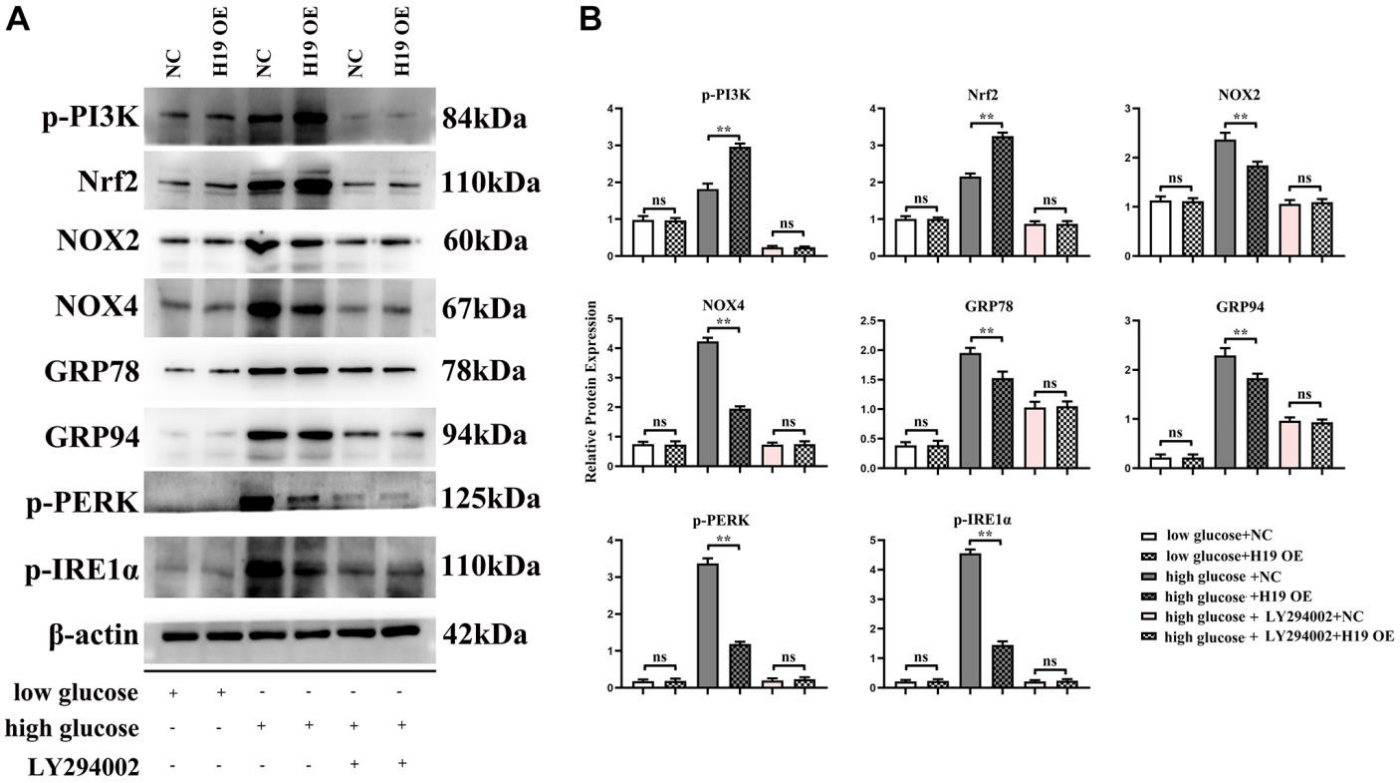
**H19 suppressed ROS, and ERS by activating PI3K-Nrf2 signals.** (**A**) Protein bands of the p-PI3K, Nrf2, NOX-2/-4, GRP78, GRP94, p-PERK, and p-IRE1a in each group. (**B**) Statistical data of each group, *n* = 3/group.

PI3K-AKT signals are key and powerful suppressor for oxidative stress/ROS and conversely, ROS inhibits activation of PI3K. Furthermore, ROS induces ERS in DCMs, [31–34, 36, 37, 45] and H19 indirectly activates PI3K by sponging miR-140-5p. Our results showed that H19 significantly increased the activation of PI3K and Nrf2 and thus decreased the expression of NOX-2/-4, as well as under high glucose conditions, and unfolded protein response’ proteins GRP-78/-94 and p-PERK and p-IRE1α, and these effects of H19 were corrected or rescued by PI3K inhibitor LY294002 (Figure 7).

## DISCUSSION

The ER and mitochondria may adjust their construction and function to respond to dynamic environmental challenges [46, 47]. Given ERS, special alterations in UPR contribute to the activation of myocardial cell apoptosis [48, 49]. Extensive studies have shown that the enlarged myocardial cell death plays a critical role in the progression of DCM [50, 51]. LncRNAs including DCM are crucial players in various physiological processes implicated in the pathogenesis of several cardiovascular diseases [52, 53]. A key lncRNA in DCM is lncRNA H19 (H19) which produces a 2.3-kb non-coding mRNA and is conserved via matriarchal evolution [23]. However, the molecular mechanism of the effect of H19 on myocardial cell apoptosis remains unknown and the relationship between RES-induced apoptosis and H19 in DCM has not been investigated. The purpose of this study was to determine the possible role and mechanism of H19 in RES-induced cardiomyocyte apoptosis in DCM. Our study’s key findings were that H19 significantly enhanced cardiac systolic performance in STZ-induced rats and that H19 administration decreased diabetes-induced myocardial fibrosis and cardiomyocyte death. Additionally, bioinformatics research was used to determine the DEGs and active pathways associated with DCM. H19 was among the downregulated DEGs. The enrichment analysis of GO and KEGG pathway showed that DEGs were enriched in PI3K-AKT signaling pathway, regulation of apoptotic process and IRE1-mediated unfolded protein response. The online database LncRRIsearch and RNARNA were used to predict the interaction between lncRNAs and mRNAs. Among the identified target mRNAs, PI3KCA(PI3K) was chosen as a potential target of lncRNA H19 and their binding site was shown. Furthermore, H19 increased the expression of ROS, ERS-associated apoptosis markers and phosphorylation of PI3K, AKT and mTOR *in vivo*, and repressed ERS and activated PI3K after HG *in vitro*.

It is a consensus that cardiomyocyte apoptosis may ultimately lead to damaged cardiac function and myocardium fibrosis [54]. Emerging evidence indicates that the upregulation of ROS, and ERS are the main reason for cardiomyocyte apoptosis in diabetes cardiomyopathy (DCM) [55]. Meanwhile, DCM is also a common consequence of diabetes, characterized by changes in heart structure and cardiomyocyte death [55, 56]. Diabetic cardiomyopathy (DCM) is characterized by early damage of diastolic function, accompanied by the development of cardiomyocyte hypertrophy and myocardial fibrosis [1]. Increasing the production of reactive oxygen species and reducing antioxidant defense make an important contribution to DCM. Under the condition of high glucose, due to abnormal glucose metabolism and impaired glucose utilization, a large number of reactive oxygen species such as MDA production increase, which directly damage the myocardium, or indirectly affect the function of sarcoplasmic reticulum calcium pump [2–4]. Enhancement of antioxidant stress response, such as SOD, can effectively reduce cardiomyocyte injury and cardiac insufficiency [5]. PI3K has dual activities of protein kinase and lipid kinase, which has been divided into type I, II and III, in which I is mainly involved in the mechanism of cardiovascular disease [6]. PI3k/Akt signaling pathways are involved in cardiomyocyte growth, metabolism and apoptosis to protect the heart and reduce myocardial hypertrophy and fibrosis [7, 8]. Extracellular ligands including Integrin, RTK (receptor tyrosine kinase), BCR (B cell receptor) and GPCR (G protein-coupled receptor) activate cell membrane surface receptors and induce intracellular PI3K to bind to them. PI3K activates PDK (Phosphoinositide-dependent protein kinase) through PIP3, which in turn activates downstream Akt [7, 8]. There are three activated pathways of PI3K/Akt, including insulin, GSK-3 and mTOR [2]. In the case of diabetes, oxidative stress was enhanced, PI3K/Akt signal was down-regulated, and myocardial damage was aggravated [9]. Blocking PI3K/Akt pathway in diabetic rats significantly reduced the expression of eNOS and mTOR proteins that regulate apoptosis [10, 11]. Activating PI3K/Akt promotes the phosphorylation of eNOS and mTOR, and eNOS further promotes the production of NO, which is a powerful inhibitor for oxidative stress, result into the protective roles in DCM from myocardial death [12]. Thus it can be seen that DCM, oxidative stress and PI3K/Akt are closely related. In the future, oxidative stress can be alleviated and inhibited by activating the PI3K/Akt pathway, thus reducing DCM myocardial injury and delaying the progression of myocardial hypertrophy and fibrosis. H19 administration significantly reduced the chamber dilation, interstitial fibrosis, and collagen deposition in myocardial tissue of DM, suggesting that H19 may repress apoptosis of cardiomyocytes and reduce cardiac fibrosis in DM.

PI3K/AKT/mTOR pathway is a critical cell signaling pathway that participates in regulating ERS-induced cell death. In the present study, PI3KCA(PI3K) was chosen as a potential target of lncRNA H19. However, the role of H19 in PI3K-induced apoptosis of cardiomyocytes has not been explored. Previous studies identified H19 combined with miR-140-5p results in the degradation of miR-140-5p [57], and miR-140-5p binds to the PI3KCA (PI3K), so the over-expression of H19 leads to the raised protein expression and these results were shown in Figure 2C, 2D, and 2F. Firstly PI3K-signal modulate the process of fibrosis by inhibiting the expression and activation of FOXO1/3a and GSK3β/ASK1 and therefore inhibited the deposition of FGF, Collagen I/III as well as a-SMA, the fibrosis contents [58], and we identified these mechanisms and shown in Figure 2E and 2F. Secondly from these points of view, we hypothesized that H19 may be associated with PI3K in DCM development and protect cardiomyocytes against ERS-induced cell apoptosis [59]. previous studies have identified that Nrf2 signaling plays a protective role in cardiovascular diseases, and moreover, the Nrf2 as well as ROS is the bridge between the PI3K signal and ERS [60–63] as well as unfolded protein reaction [58, 64, 65], thus in this research we detected the increased Nrf2 expression consistent with H19 OE induced the raised expression and activation of PI3K signal and decreased Unfolded protein reaction and ERS associated proteins of GRP 78/94, p-PERK, p-IRE1α, ATF6, CHOP, cleaved caspase-3, cleaved caspase-9, cleaved caspase-12, and BAX was significantly enhanced in cardiac tissues in DCM mice, while H19 administration reversed the up-regulation of these proteins. These findings implied that H19 reduces HG-induced ERS-associated myocardial apoptosis *in vivo* and *in vitro* experiments.

Several previous studies highlight that an ERS-induced increase of CHOP may trigger cell apoptosis and play a critical role in regulating the fate of cells under ERS. In the *in vitro* experiments, high glucose (HG)-induced HL-1 cells were used to establish the inflammation model. HL-1 cells were transfected to lentivirus pcDNA-H19, after that, these cells were stimulated with or without HG for 24 h. Immunofluorescence staining also showed that HG stimulation significantly upregulated the expression of p-PERK and CHOP in HL-1 cells, whereas H19 markedly reversed the HG-induced downregulation of p-PERK and CHOP. Thapsigargin is a potent ERS inducer that has been successfully used in a variety of cell lines. CHOP and p-PERK expressions were significantly increased in HL-1 cells treated with Thapsigargin. These findings suggested that H19 may protect cardiomyocytes from ERS when exposed to HG. Furthermore, our Western blotting further confirmed that H19 attenuates ROS, ERS-induced cardiac apoptosis and activates the PI3K signaling.

**Figure 8:**
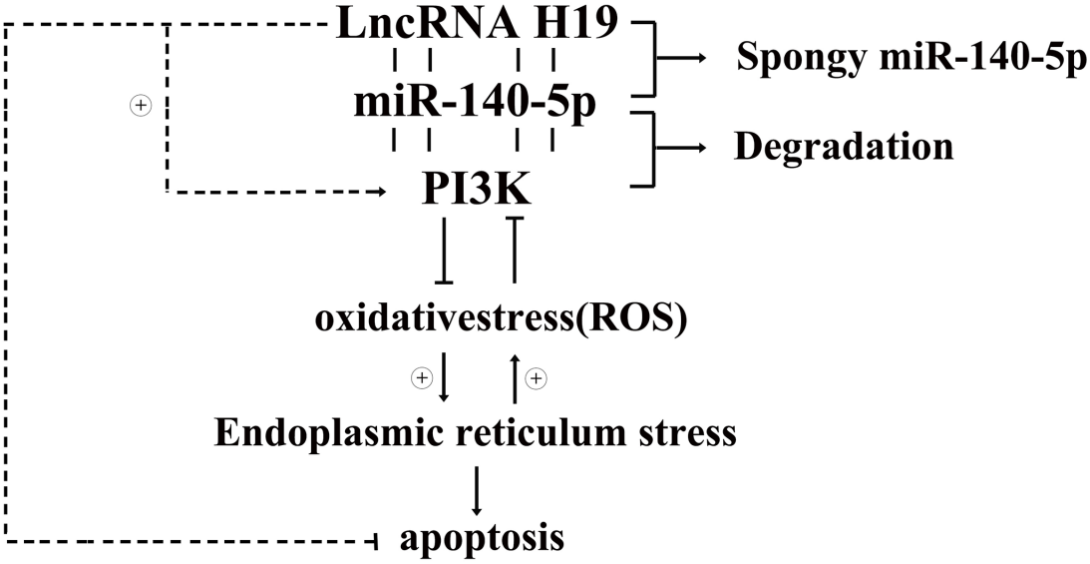
Diagrammatic sketch for lncRNA H19’s protective roles in DCMs by activating PI3K/AKT signals and inhibiting ROS, ERS induced apoptosis.

In conclusion, lncRNA H19 may protect against DCM by inhibiting ROS, and RES-associated cardiac apoptosis through PI3K/Akt/mTOR signaling pathway. Additionally, H19 plays an essential role in enhancing ROS, and ERS-associated cardiomyocyte apoptosis. Comprehending the practical role of ERS-associated cardiac apoptosis in the pathogenesis of DCM is a talented way regarding the improvement of more targeted treatments. This work suggests that PI3K/AKT/mTOR mediated H19’s protective impact against ERS-associated apoptosis and highlights the potential for H19 to prevent cardiac damage and the development of DCM. This study establishes novel evidence for H19’s protective effects and identifies novel therapeutic targets for patients with DCM and the associated mechanisms were showed in Figure 8.

## Supplementary Materials

Supplementary Figures
