# Supplementary material for: LncRNA H19 inhibits ER stress induced apoptosis and improves diabetic cardiomyopathy by regulating PI3K/AKT/mTOR axis
Source: Aging (Albany NY). 2022 Aug 30;14(16):6809–28. doi: 10.18632/aging.204256 (PMC9467416; doi:10.18632/aging.204256)
Supplement: Supplementary Figures [file aging-14-204256-s001.pdf]

SUPPLEMENTARY FIGURES

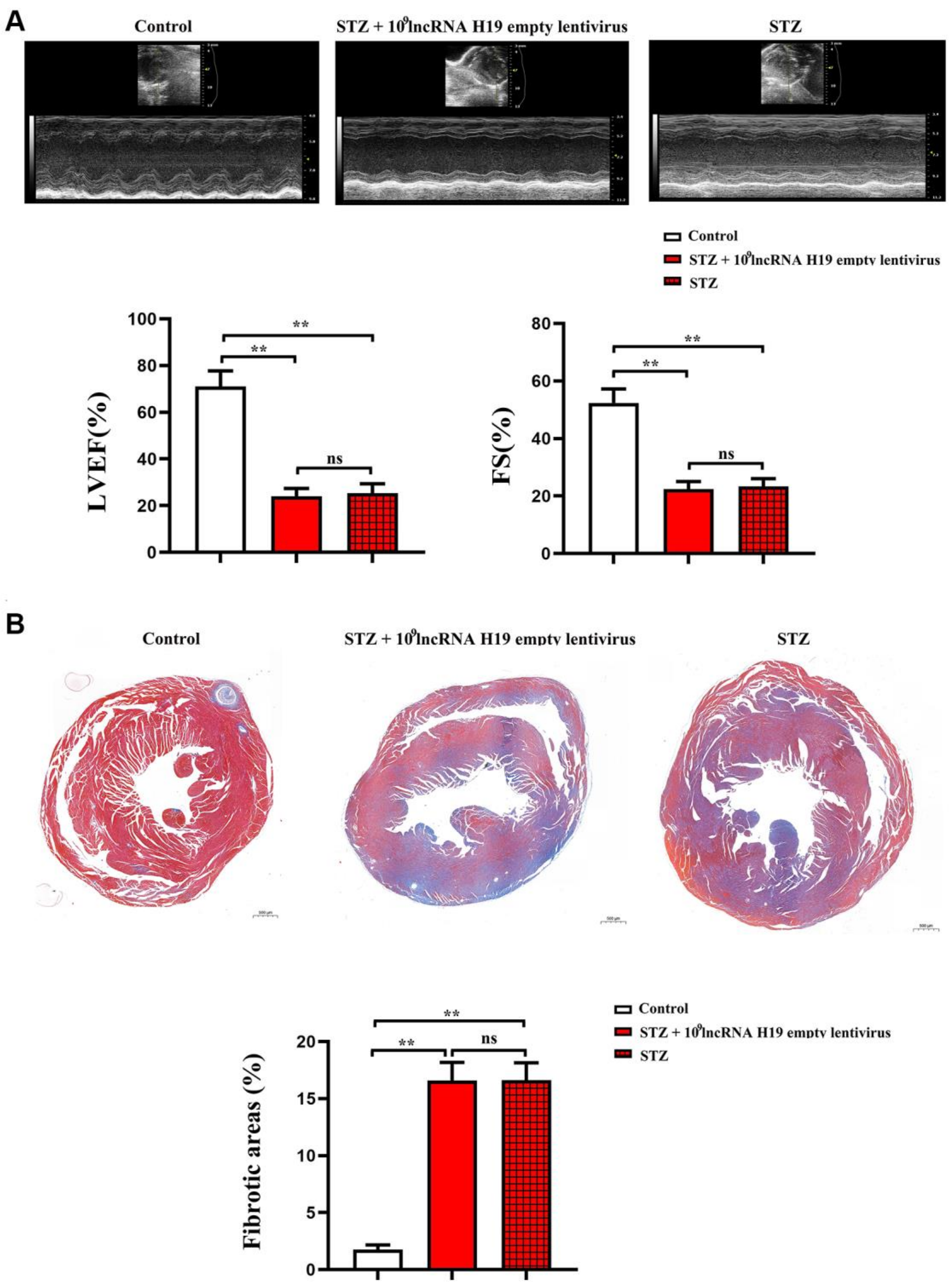

Supplementary Figure 1. LncRNA H19 empty lentivirus had no effects on the progression of DCM. (**A**) There were no significant differences in left ventricular function in STZ + LncRNA H19 empty lentivirus and STZ group, and the 2 groups showed significant decreased LVEF and FS vs. control group. (**B**) The ratio of fibrotic areas in whole heart were no significant differences in STZ + LncRNA H19 empty lentivirus and STZ group, and the 2 groups showed notable raised ratio of fibrotic areas in heart vs. control group. Data are expressed as mean ± SEM. $^{*}P < 0.05$, $^{**}P < 0.01$. STZ+LncRNA H19 empty lentivirus, and STZ group vs. control group, n = 6/group.

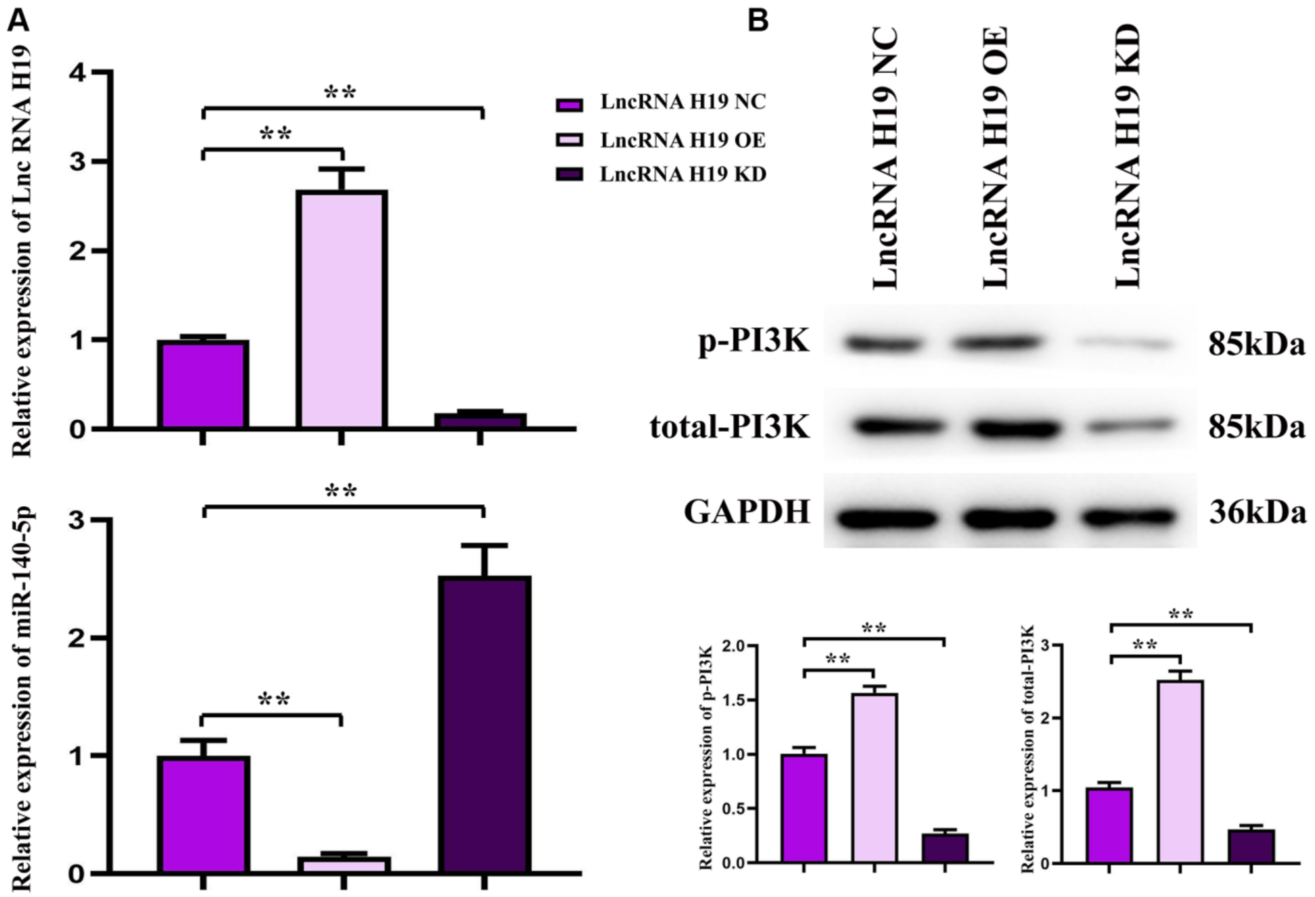

Supplementary Figure 2. The relationship between lncRNA H19, miR-140-5p and PI3K. (**A**) The relative expression levels of lncRNA H19 and miR-140-5p levels in HL-1 cells after infected with lncRNA H19 NC, OE, KD lentivirus. (**B**) The phosphorylated and total protein levels of PI3K after infecting with lncRNA H19 NC, OE, KD lentivirus. Data are expressed as mean ± SEM. $^{*}P < 0.05$, $^{**}P < 0.01$. LncRNA H19 NC group vs. OE and KD group, $n = 3$/group.
